# Novel biallelic variants in *COL7A1* cause recessive dystrophic epidermolysis bullosa

**DOI:** 10.1002/mgg3.1347

**Published:** 2020-06-14

**Authors:** Neng Yang, Yongyi Ma, Hong Yao, Qing Chang, Victor Zhang, Zhiqing Liang, Xiongwei Cai

**Affiliations:** ^1^ Key Laboratory of Freshwater Fish Reproduction and Development Ministry of Education Laboratory of Molecular Developmental Biology School of Life Sciences Southwest University Chongqing China; ^2^ Department of Gynecology & Obstetrics Southwest Hospital Third Military Medical University (Army Medical University) Chongqing China; ^3^ Department of Human and Molecular Genetics Baylor College of Medicine Houston TX USA; ^4^ AmCare Genomics Lab Guangzhou China

## Abstract

**Background:**

Autosomal recessive dystrophic epidermolysis bullosa (RDEB) is an incurable and severe inherited skin disorder characterized by recurrent blistering at the sublamina densa beneath the cutaneous basement membrane. It is caused by biallelic loss‐of‐function mutation in the gene encoding type VII collagen (*COL7A1*). This study aimed to identify the causative variants of a Chinese RDEB patient and further provide prenatal diagnosis for the ongoing risk pregnancy of the proband's mother.

**Methods:**

Clinical exome sequencing (CES) has been performed and an in‐house pipeline was used to conduct a phenotype‐driven data analysis. A minigene assay was used to verify the pathogenicity of a novel splice site variant in the *COL7A1*.

**Results:**

Here we report two compound heterozygous variants in *COL7A1*, c.3867delT (p.G1290Efs*35) and c.5532+4_5532+5delAG, identified in a RDEB patient by CES. The minigene assay confirmed that thec.5532+4_5532+5delAGchange was a noncanonic splice site variant leading to in an in‐frame deletion of exon 64. Prenatal diagnosis indicated that the present pregnancy of the patient's mother was not affected.

**Conclusion:**

Our study expands the mutation spectrum of *COL7A1* and demonstrated that CES and minigene assays were efficient tools for RDEB molecular diagnoses.

## Graphical abstract

Our study expands the mutation spectrum of *COL7A1* and demonstrated that clinical exome sequencing and minigene assays were efficient tools for recessive dystrophic epidermolysis bullosa molecular diagnoses.

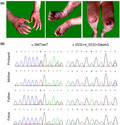

## 1 INTRODUCTION

Epidermolysis bullosa (EB) is a group of inherited skin conditions characterized by the loss of skin integrity that leads to blisters and erosions with little or no trauma. More than 20 genes associated with dermal–epidermal junction assembly are involved in this disease. As the phenotype highly variable, EB was originally classified into four major types (simplex, junctional, dystrophic, and Kindlersyndrome) and further subclassified in multiple clinical subtypes based on the level of blister formation in the cutaneous basement membrane zone and the underlying molecular etiology (Fine et al., 2014; Uitto, Bruckner‐Tuderman, McGrath, Riedl, & Robinson, 2018). In addition, recent perspective proposes that EB may be classified in nonsyndromic and syndromic forms according to with or without extracutaneous clinical manifestations (Vahidnezhad, Youssefian, Saeidian, & Uitto, 2019). Recessive dystrophic epidermolysis bullosa (RDEB) is one of the most severe nonsyndromic forms, which is caused by biallelic loss‐of‐function variants in the collagen type VII gene (*COL7A1*, OMIM accession number: 120,120) (Wagner et al., 2010). In the present study, we identified two previously unreported recessive pathogenic variants of *COL7A1* in an RDEB patient by using clinical exome sequencing (CES) and minigene assay.

## 2 SUBJECTS AND METHODS

### 2.1 Editorial policies and ethical considerations

This work was permitted by the ethics committee of Southwest Hospital, Third Military Medical University. The informed consent was obtained from patient for molecular study and publication.

### 2.2 Clinical report

**FIGURE 1:**
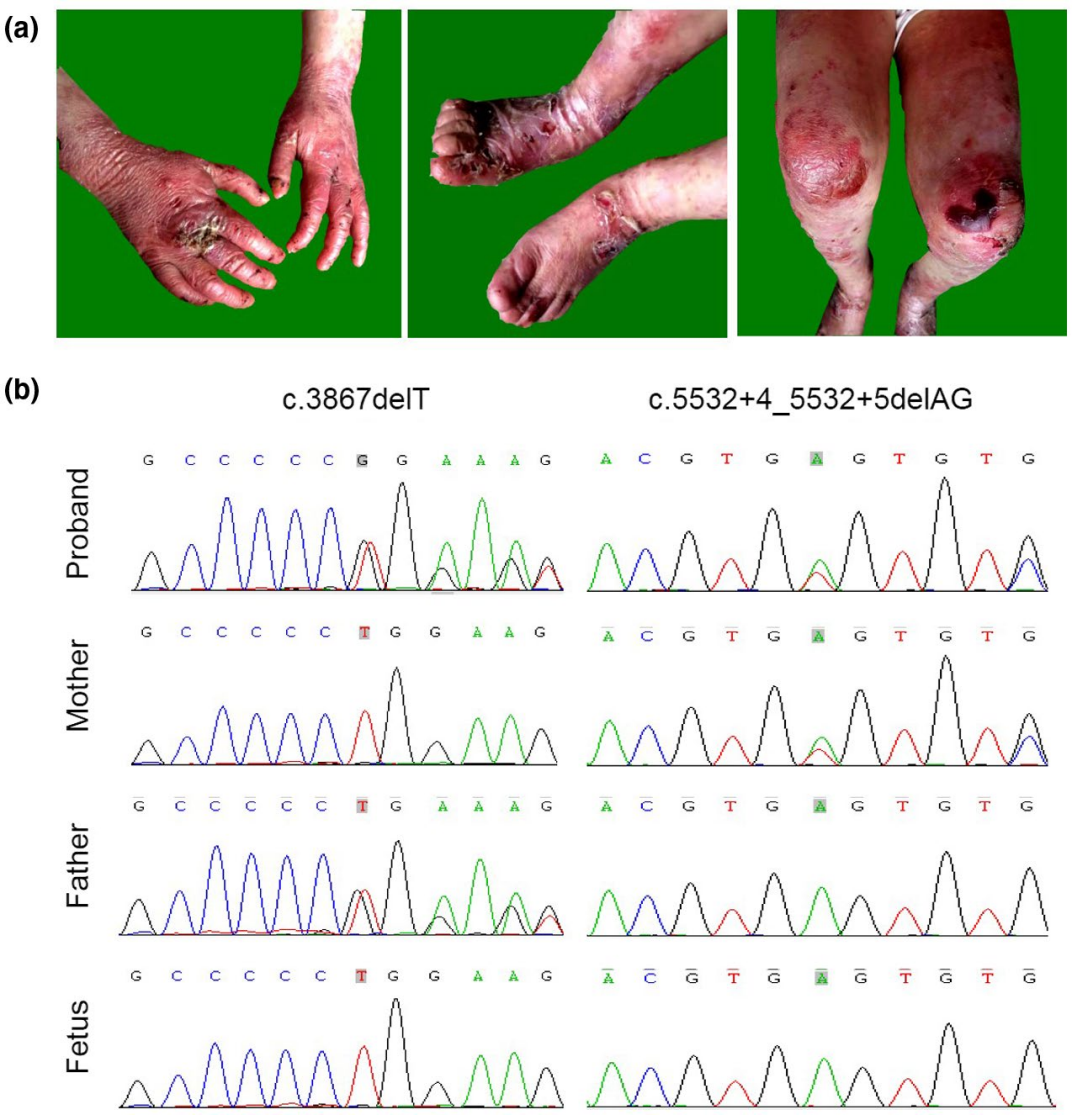
Feature of the patient and the *COL7A1* mutation in this family. (a) Clinical manifestations of the proband. Blisters, blood blisters, and scarring formation on the hands (left panel), feet (middle panel), knees, and tibialis (right panel). (b) Pedigree analysis and prenatal diagnosis. The proband inherited c.3867delT and c.5532+4_5532+5delAG from her parents and neither variants were detected in the fetus

The patient was a 3‐year‐old Chinese Han girl who was the first child of an unrelated healthy couple. As birth certificate recording, the patient was born with weight 3.1 kg (25–50th percentile), height 48 cm (25–50th percentile), and head circumference 34 cm (50th percentile). The weight was 11 kg (3rd percentile), height 87 cm (3rd percentile), and head circumference 48 cm (25–50th percentile) at her 3 years. Her parents reported that she was noted skin absence of the lower left limb at birth, and had been suffering from slight trauma‐induced blisters, blood blisters, scarring, and milia formation on the trunk, extremities, and neck since birth. Physical examination at the clinic shows that the skin lesion was particularly severe on her hands, feet, elbows, tibialis, and knees. She also had slightly webbed toes (but fingers unaffected), and dystrophic nails. Tooth decay and dental loss, and oral mucosal blood blisters were also detected (Figure 1a). Frequent melena and hematochezia were reported by the parents indicating that gastrointestinal mucosa injury was involved. The patient had normal facial feature, intelligence, speech, and vision, and no skeletal abnormality was observed. Abdominal and cardiac ultrasound assessment was not carried out due to the refusal of the parents. Skin biopsies were not performed for the same reason. The parents are healthy as neither of them had any signs indicating EB, including blistering, scarring, or nail dystrophy.

The parents had been told by a dermatologist that the patient might be involved in dystrophic epidermolysis bullosa (DEB), although no genetic testing was performed. Because the patient's mother had been pregnant for 8 weeks at the first visit, both of proband's gene diagnosis and prenatal diagnosis were expected to be provided.

### 2.3 Genetic analyses

We performed CES for the proband. Library preparation, in‐solution hybridization exome capture, next‐generation sequencing, and data analyses were conducted as previously described (Wang et al., 2019). Candidate causative variants identified in the proband were confirmed by Sanger sequencing. Parents were also tested to evaluate the mode of inheritance.

### 2.4 Minigene assay

The target genomic fragment of *COL7A1* (GenBank, NG_007065.1, NM_000094.3) encompassing exons 62–66 was amplified by PCR using the proband's genomic DNA as a template. The PCR primers included restriction enzyme sites for HindIII (P1: 5′AAaagcttGGTGCTGCAGGCAAA) and EcoRI (P2: 5′AAgaattcTTCTCTCCCAGAGGC). PCR products were then cloned into plasmid pcDNA3.0. Positive clones were sequenced to exclude the presence of additional variants and to distinguish between the wild‐type or specific variants found in the proband. Transfection was performed using the LipoFiterTM 3.0 Liposomal Transfection Reagent (Hanbio) according to the manufacturer's instructions. Wild‐type and mutant constructs were transiently transfected into HeLa cells, respectively, and pcDNA3.0 without inserts as a blank control. Total RNAs were isolated from transfected cells by the QIAzol Lysis Reagent (Qiagen) and reverse transcribed using a PrimeScript™ RT reagent Kit with gDNA Eraser (Takara). RT‐PCR was performed using the P3 (5′‐GGGCTTCCAGGCCTCCGTGGA) and P4 (5′‐CTTCCTCCCGTCTTCTCCAGG) located in exon 63 and exon 65 of *COL7A1*, respectively. RT‐PCR products were separated by electrophoresis in a 2% agarose gel. The fragments were purified and inserted into a plasmid T‐vector (Tsingke) according to the manufacturer's instructions for Sanger sequencing.

## 3 RESULTS

### 3.1 Molecular findings

Two compound heterozygous variants in *COL7A1 *comprising c.3867delT in exon 31 and c.5532+4_5532+5delAG in intron 64 were identified by CES and confirmed by Sanger sequencing (Figure 1b). Both variants were extremely rare and have not been reported in previous literature nor detected in the whole genome or exome sequencing public (1,000 genomes, gnomAD, and GO‐ESP) and in‐house databases. The c.3867delT, inherited from the father, is a deleterious variant leading to a frameshift and premature stop codon after 35 amino acids (p.G1290Efs*35). The c.5532+4_5532+5delAG, inherited from the mother, is adjacent to the donor splicing site of intron 64, which was predicted to result in the disruption of a 5' donor splice site by the Human Splicing Finder (http://umd.be/HSF3/credits.html) (with a ΔCV score −10.13).

### 3.2 Splicing assays

**FIGURE 2:**
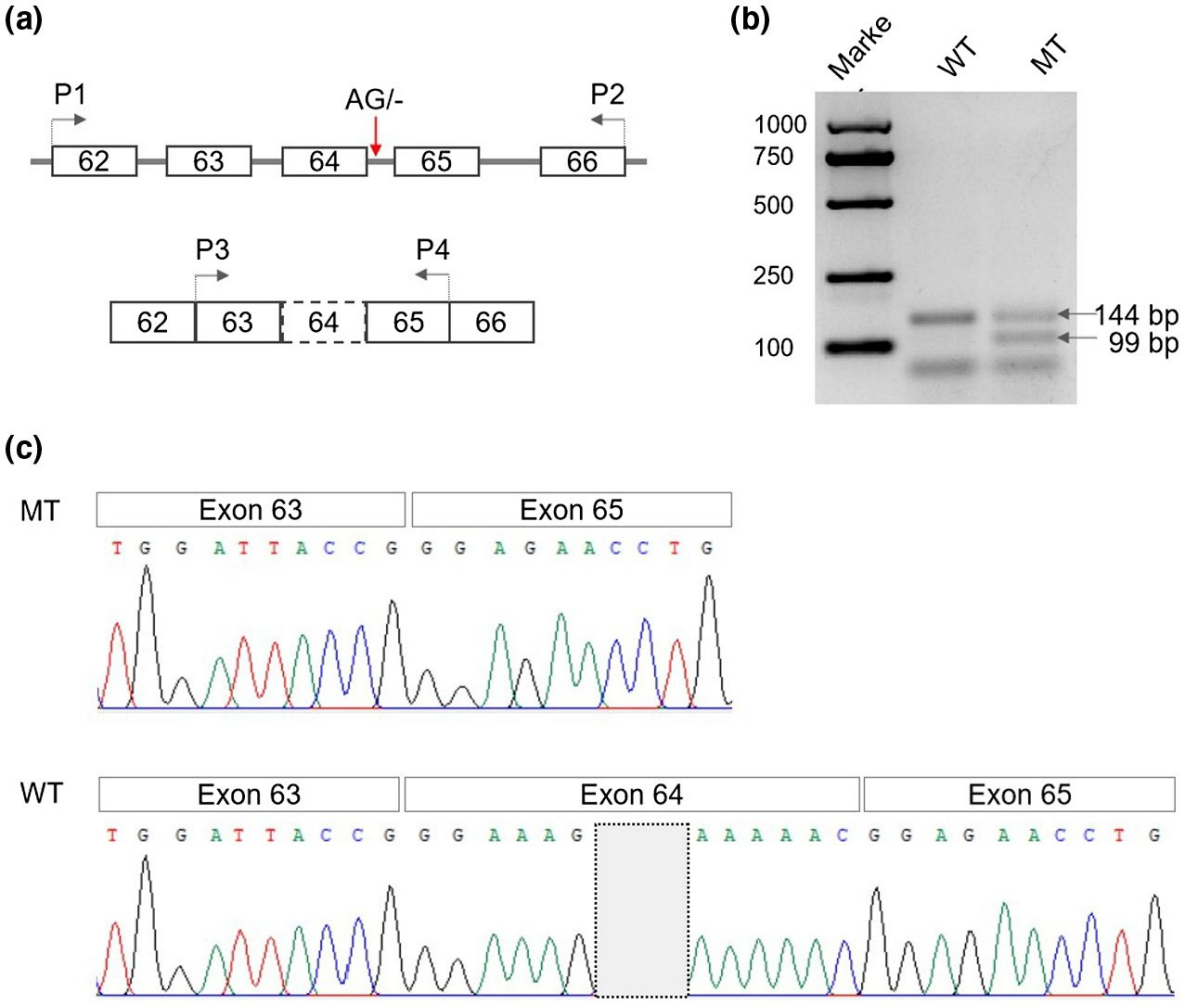
In vitro minigene assay. (a) Schematic of minigene assay, primers P1/P2 were used to amplify target genomic fragment encompassing exon 62 to exon 66, primers P3/P4 were used for RT‐PCR, the red arrow indicated the variant of c.5532+4_5532+5delAG in intron 64. (b) Agarose gel electrophoresis of RT‐PCR products, the longer band represented the wild‐type transcript (WT) with a length of 144 bp, the shorter band represented the mutated transcript (MT) with a length of 99 bp, the bottom bands are primer dimer. (c) Sanger sequencing of the RT‐PCR products, the wild‐type transcript consisted of exon 63, 64, and 65, in contrast, the mutated transcript consisted of exon 63 and 65, without exon 64

Since the patient's RNA was not available, we performed an in vitro minigene assay to functionally validate the effect of c.5532+4_5532+5delAG on splicing. RT‐PCR analysis using the wild construct transinfected cells showed one amplicon. In contrast, the mutant construct showed an additional shorter amplicon, suggesting aberrant splicing (Figure 2b). To characterize the nucleotide change underlying this event, we further purified and sequenced the RT‐PCR products. The results showed that the larger amplicon consisted of exon 63 (63 bp), 64 (45 bp), and 65 (36 bp), with a length of 144 bp, corresponding to the correctly spliced transcript. However, the shorter amplicon contained only exon 63 and 65, with a length of 99 bp (Figure 2c). This result indicated that the c.5532+4_5532+5delAG variant resulted in the skipping of exon 64, leading to an in‐frame deletion of 15 amino acids. Previous studies had demonstrated that the skipping of exon 64 was a causative variant (Horev et al., 2003; Huang, Wong, & Burd, 2011). Therefore, based on the splicing test and findings in other reports, c.5532+4_5532+5delAG was classified as pathogenic.

### 3.3 Prenatal diagnosis

Since pathogenicity of both variants validated in this family, amniocentesis and prenatal gene testing were performed at 19 weeks of another pregnancy. By targeted Sanger sequencing, neither of the two variants were found in the fetus (Figure 1b). Postnatal follow‐up confirmed that the neonatal was unaffected with RDEB.

## 4 DISCUSSION

Dystrophic epidermolysis bullosa, featured with defective anchoring fibrils and consequent separation of the sub‐basal lamina, is caused by variants in the *COL7A1* in either autosomal dominant or autosomal recessive fashion (Christiano et al., 1994; Fine et al., 2014). The diagnosis of DEB is based on clinical findings and the identification of causative variants in *COL7A1*. Genetic testing is necessary prenatally in families with affected members. The clinical phenotypes of DEB show considerable clinical variability and may overlap with other types of EB. Therefore, when molecular genetic testing is required, it is appropriated to apply more comprehensive genomic sequencing approaches (Gong, Liu, Li, & Xu, 2018; Has & Fischer, 2018; Lin et al., 2012; Mahajan et al., 2018).

The *COL7A1* contains 118 exons, coding for the alpha‐1 chain of type VII collagen composed of 2,944 amino acids. Type VII collagen functions as an anchoring fibril between the external epithelia and the underlying stroma. The protein consists of three distinctive structure domains: triple‐helical collagenous (THC) domain encoded by exons 29–112, an amino terminus (NC‐1) encoded by exons 2–28, carboxyl terminus (NC‐2) encoded by exons 113–118 (Christiano et al., 1994; Lin et al., 2012).

In our patient, two compound heterozygous variants in *COL7A1* were identified by CES and confirmed by Sanger sequecing. The paternal variant was a 1‐bp deletion in exon 31 (c.3867delT), leading to a reading frameshift and premature termination codon (PTC) after 35 amino acids (p.G1290Efs*35). The consequence is the truncation of 1654 amino acids downstream exon 31, which include part of THC and complete NC‐2. The maternal variant was a 2‐bp deletion at position the +4 and +5 positions in intron 64 (c.5532+4_5532+5delAG). As the reference sequence at the 5′ end of intron 64 is a typical donor splice site consensus sequence for U2 introns (GTRAGT), this variant was unsurprisingly predicted to damage the function of the 5′ donor splice site resulting aberrant splicing (Zhang, 1998). However, the definition of a noncanonical splicing variant, based on silico predictions alone, is not robust for clinical diagnoses. Since the patient's RNA was not available, we conducted* *a minigene assay to clarify the effect of this change on splicing. The results indicated a complete in‐frame skipping of exon 64 (15 amino acids) within the THC domain of the type VII collagen polypeptides. It was noteworthy that two variants of single nucleotide substitution at positions +4 (c.5532+4A>G) and +5 (c.5532+5G>A) in intron 64 of *COL7A1* had been established as RDEB causative variants in two previous unrelated studies (Horev et al., 2003; Huang et al., 2011). One of the studies analyzed the specific outcome of both changes at the RNA and protein levels in a patient with c.5532+5G>A and another PTC variant in compound heterozygous status. The analysis of the patient's RNA demonstrated that the c.5532+5G>A resulted in complete in‐frame skipping of exon 64 within the THC region of the type VII collagen polypeptides. The protein analysis by immunofluorescence staining showed undetectable expression of type VII collagen polypeptides at the dermal–epidermal junction in the skin biopsy (Huang et al., 2011). Our research findings, together with previous reported experimental evidence, suggested that in‐frame skipping of exon 64 is a loss of function mutation which will disrupt the production, secretion, and anchoring of the fibril assembly of type VII collagen.

Dystrophic epidermolysis bullosa has wide clinical spectrum but commonly severe. Although recent advances in therapeutics research, it is still incurable and profoundly affects functional abilities and quality of life till today. Early diagnosis is important to prevent and/or minimize tissue damage via appropriate treatment and management (El Hachem et al., 2014; Uitto, 2019; Uitto et al., 2018; Venti et al., 2020). On the other hand, due to concerns about the risk of recurrence, most of the patients families prefer to have healthy offspring through prenatal diagnosis or preimplantation diagnosis. Identification of causative variant(s) of the proband is a prerequisite for prenatal diagnosis in families at risk for recurrence of EB. For ongoing risk pregnancies in which the molecular etiology of the proband is unclear, rapid gene diagnosis is vital and urgent. Our study demonstrated that exome sequencing is a rapid and efficient diagnostic strategy for DEB. In addition, we also demonstrated that the minigene assay was a reliable and easily applicable tool for evaluating the splicing effect of *COL7A1* atypical splice variants.

## CONFLICT OF INTEREST

The authors declare that they have no conflict of interest.

## AUTHOR'S CONTRIBUTION

Yongyi Macollected the samples and designed the study. Neng Yang conducted the research, and analyzed the results. Neng Yang and Yongyi Mawrote the paper. Hong Yao, Qing Chang, and Victor Zhangrevised the paper. Zhiqing Liang and Xiongwei Cai initiated and organized the study.

## Data Availability

Data sharing are not applicable to this article as no new data were created or analyzed in this study.
